# Dissociation and Brain Rhythms: Pitfalls and Promises

**DOI:** 10.3389/fpsyt.2021.790372

**Published:** 2021-12-03

**Authors:** Tineke Grent-'t-Jong, Lucia Melloni, Peter J. Uhlhaas

**Affiliations:** ^1^Department of Child and Adolescent Psychiatry, Charité Universitätsmedizin, Berlin, Germany; ^2^Institute of Neuroscience and Psychology, University of Glasgow, Glasgow, United Kingdom; ^3^Department of Neurology, New York University School of Medicine, New York, NY, United States; ^4^Department of Neuroscience, Max Planck Institute for Empirical Aesthetics, Frankfurt Am Main, Germany

**Keywords:** dissociation, ketamine, neural oscillations, optogenetics, animal models, translational neuroscience

## Abstract

Recently, Vesuna et al. proposed a novel circuit mechanism underlying dissociative states using optogenetics and pharmacology in mice in combination with intracranial recordings and electrical stimulation in an epilepsy patient. Specifically, the authors identified a posteromedial cortical delta-rhythm that underlies states of dissociation. In the following, we would like to critically review these findings in the context of the human literature on dissociation as well as highlight the challenges in translational neuroscience to link complex behavioral phenotypes in psychiatric syndromes to circumscribed circuit mechanisms.

Dissociation is a state of mind in which there is a disconnection between a person's thoughts, memories, feelings, actions, and/or sense of self. Dissociation can include symptoms of derealization (e.g., feeling disconnected from one's surroundings), depersonalization (e.g., feeling disconnected from one's self), and often also amnesia, identity confusion, or identity alteration, which occur frequently in psychiatric disorder, such as post-traumatic stress disorder, psychosis, and dissociative identity disorder. Dissociative states can also be elicited by pharmacological manipulations and are a signature of neurological conditions, such as epilepsy.

Recently, Vesuna et al. (1) proposed a novel circuit mechanism underlying dissociative states. The authors administered ketamine, a dissociative anesthetic, to mice and obtained electrophysiological recordings from several brain regions, including retrosplenial cortex. Dissociation-like behavior was operationalized as intact reflexive paw-flicking, indexing stimulus detection, but absent paw-licking, signaling abolished affective responses, when placing the animals on a 55°C surface (hot plate test). Vesuna et al. observed that ketamine selectively reduced only paw-licking which was correlated with a 1–3 Hz rhythm in posteromedial cortex that was furthermore selectively coupled to thalamic circuits. Moreover, optogenetic activation of layer 5 neurons in retrosplenial cortex at delta frequencies recreated this behavioral phenotype. Remarkably, a comparable 3 Hz rhythm in posteromedial cortex, which correlated with pre-seizure aura and self-reported dissociation, was observed in a patient with focal epilepsy and electrical stimulation of the posteromedial cortex induced dissociative experiences.

The study of Vesuna et al. (1) is a tour-de-force, impressively linking cell-type specific recordings and optogenetics in mice with invasive cortical recordings in humans to identify a putative mechanism for dissociative states. However, in regards to the central conclusion that delta-oscillations in the posteromedial cortex underlie dissociative experiences, we would like to suggest some potential caveats and alternative explanations.

One prediction following the findings by Vesuna et al. is that circumscribed delta-band oscillations in posteromedial cortex are a common mechanism underlying dissociative states in psychiatric disorders as well as during administration of dissociative drugs, such as ketamine. Yet, preliminary electrophysiological data in humans offer a somewhat complex picture. First, ketamine-induced dissociative experiences have been shown to correlate with brain-wide alpha power changes, rather than changes in constrained, delta-oscillations (2). Furthermore, in patients with dissociate experiences, increased EEG resting-state power in the theta range correlate with both increased state- and trait-like dissociative experiences (3). Moreover, in contrast to the rather local abnormality in posterior medial cortex predicted by Vesuna et al. study, fMRI studies (4) have highlighted an extended network of dorsomedial and dorsolateral prefrontal cortex, bilateral superior frontal regions, (anterior) cingulate, posterior association areas, and basal ganglia underlying dissociative states.

Critically, Vesuna et al. (1) observed that only administration of N-Methyl-D-aspartic acid or N-Methyl-D-aspartate receptor antagonists, such as ketamine or PCP, but not other analgesic, anesthetic, sedative, or hallucinogenic drugs, were specifically associated with delta-oscillations in the posteromedial cortex. However, increased delta-oscillations were only found for higher dosages starting at 25 mg/kg body weight, which corresponds to approximately 2–3 mg/kg in human subjects. In human studies, however, lower dosages (<1 mg/kg) are typically used to induce dissociative experiences, mostly to avoid more severe side effects (e.g., nausea) and/or (partial) loss of consciousness. Accordingly, one possibility is that the administration of higher dosages in mice, used to create measurable dissociative states, also lead to changes in the level of consciousness.

This is an important consideration in light of the role of the posteromedial 3 Hz rhythm during changes in consciousness in epilepsy. Narrow 3–4 Hz delta-band oscillatory responses have been previously associated with absence seizures, brief (up to 10 s) losses of or decrements in the level of consciousness, with posteromedial cortex activation starting already during the aura state (5). Interestingly, a model has been proposed (6, 7) in which seizure activity (e.g., originating from temporal regions) first spreads to midline subcortical structures, including posteromedial cortex, before leading to decreased activity in frontal-parietal association cortex and an associated loss of consciousness. Accordingly, a possible alternative explanation for the findings by Vesuna et al. could be that delta-band oscillations indexed a change in the level of consciousness, which was accompanied or preceded by dissociative experiences. In this context, it also important to note that electrical stimulation in an epilepsy patient was applied at 50 Hz but not at delta-frequencies, suggesting that delta-frequency modulation was not necessary for dissociative experiences.

In conclusion, Vesuna et al. provide a fascinating account of how delta-oscillations in the posteromedial cortex underlie complex changes in behavior and possibly consciousness. However, in regards to their central conclusion that a posteromedial 3 Hz rhythm underlies dissociative states, further work is necessary to confirm this link, which could then be used for interventions targeting this area, for example, through brain stimulation.

The current findings also highlight the challenges in translational neuroscience to link complex behavioral phenotypes in psychiatric syndromes to circumscribed circuit mechanisms. Given that the current categories of psychopathological phenomena are likely to subsume heterogeneous groups of symptoms, with distinct psychological and biological origins, very specific hypothesis need to be formulated to establish casual links between neurobiological mechanisms and such phenomena. However, given the dearth of significant advances in the treatment of major mental disorders, Vesuna et al.'s seminal study opens up exciting possibilities to mechanistically dissect complex psychopathological phenomena and their neurobiological underpinnings.

## Data Availability Statement

The original contributions presented in the study are included in the article/supplementary material, further inquiries can be directed to the corresponding author/s.

## Author Contributions

All authors listed have made a substantial, direct, and intellectual contribution to the work and approved it for publication.

## Conflict of Interest

The authors declare that the research was conducted in the absence of any commercial or financial relationships that could be construed as a potential conflict of interest.

## Publisher's Note

All claims expressed in this article are solely those of the authors and do not necessarily represent those of their affiliated organizations, or those of the publisher, the editors and the reviewers. Any product that may be evaluated in this article, or claim that may be made by its manufacturer, is not guaranteed or endorsed by the publisher.
